# Evaluation of pre- and post-pyriform plasty nasal airflow^☆^

**DOI:** 10.1016/j.bjorl.2017.03.013

**Published:** 2017-05-06

**Authors:** Oscimar Benedito Sofia, Ney P. Castro Neto, Fernando S. Katsutani, Edson I. Mitre, José E. Dolci

**Affiliations:** Faculdade de Ciências Médicas da Santa Casa de São Paulo, São Paulo, SP, Brazil

**Keywords:** Nasal obstruction, Rhinomanometry, Acoustic rhinometry, Obstrução nasal, Rinomanometria, Rinometria acústica

## Abstract

**Introduction:**

Nasal obstruction is a frequent complaint in otorhinolaryngology outpatient clinics, and nasal valve incompetence is the cause in most cases. Scientific publications describing surgical techniques on the upper and lower lateral cartilages to improve the nasal valve are also quite frequent. Relatively few authors currently describe surgical procedures in the piriform aperture for nasal valve augmentation. We describe the surgical technique called pyriform plasty and evaluate its effectiveness subjectively through the NOSE questionnaire and objectively through the rhinomanometry evaluation.

**Objective:**

To compare pre- and post-pyriform plasty nasal airflow variations using rhinomanometry and the NOSE questionnaire.

**Methods:**

Eight patients submitted to pyriform surgery were studied. These patients were screened in the otorhinolaryngology outpatient clinic among those who complained of nasal obstruction, and who had a positive response to Cottle maneuver. They answered the NOSE questionnaire and were submitted to preoperative rhinomanometry. After 90 days, they were reassessed through the NOSE questionnaire and the postoperative rhinomanometry. The results of these two parameters were compared pre- and postoperatively.

**Results:**

Regarding the subjective measure, the NOSE questionnaire, seven patients reported improvement, of which two reported marked improvement, and one patient reported an unchanged obstructive condition. Regarding the rhinomanometry assessment, of 96 comparative measurements between the preoperative and postoperative periods, we obtained 68 measurements with an increase in nasal airflow in the postoperative period, 26 negative results, and two cases that remained unaltered between the preoperative and postoperative periods.

**Conclusion:**

When analyzing the results obtained in this study, we can conclude that the piriform plasty surgical procedure resulted in nasal airflow improvement in most of the obtained measurements.

## Resumo

**Introdução:**

A obstrução nasal é queixa frequente nos ambulatórios de otorrinolaringologia, e a incompetência da válvula nasal é responsável em grande parte dos casos. É bastante frequente também as publicações de trabalhos científicos descrevendo técnicas cirúrgicas sobre as cartilagens laterais superiores e inferiores para melhorar a válvula nasal. Relativamente poucos autores descrevem atualmente procedimentos cirúrgicos na abertura piriforme para incremento da válvula nasal. Descrevemos a técnica cirúrgica chamada piriformeplastia e avaliamos a sua eficácia de forma subjetiva através do questionário NOSE e de forma objetiva através do exame rinomanometria.

**Objetivo:**

Comparar as variações do fluxo aéreo nasal pré e pós-piriformeplastia através da rinomanometria e do questionário NOSE.

**Método:**

Foram estudados 8 pacientes que foram submetidos à piriformeplastia. Estes pacientes foram triados no ambulatório de otorrinolaringologia, pacientes estes que se queixavam de obstrução nasal, e que apresentavam resposta positiva a manobra de Cottle. Responderam ao questionário NOSE e foram submetidos a rinomanometria no pré-operatório. Após 90 dias foram reavaliados pelo questionário NOSE e pela rinomanometria pós-operatória. Os resultados desses dois parâmetros foram comparados pré e pós-operatoriamente.

**Resultados:**

Em relação a medida subjetiva, questionário NOSE, sete pacientes referiram melhora, sendo que dois deles referiram melhora acentuada, e um paciente referiu quadro obstrutivo inalterado. Em relação ao exame rinomanometria, de 96 medidas comparativas entre o pré e o pós-operatório, obtivemos 68 medidas com incremento ao fluxo aéreo nasal no pós-operatório, 26 resultados negativos, e dois casos inalterados entre pré e pós-operatório.

**Conclusão:**

O procedimento cirúrgico piriformeplastia conferiu melhora do fluxo aéreo nasal na maioria das medidas obtidas.

## Introduction

Nasal obstruction is a common complaint in the general population. It is defined as a discomfort characterized by the feeling of insufficient airflow through the nose. The sensation of airflow obstruction through the nose can be one of the most severe symptoms of nasal disease. The degree of nasal obstruction causing symptoms is determined not only by the severity of the obstruction, but also by the subjective perception of nasal airflow obstruction.^1^

The nose, being the upper airway entrance along with its multiple functions, such as the airflow trajectory, a chemical sensor, and air conditioner, is the first line of defense against infections. In humans and mammals, the nose is divided into two distinct anatomical pathways, and each has its own blood supply and innervation. The nasal septum divides the nose into two cavities and these consist of a bony portion and a cartilaginous portion. The lateral wall of each of these cavities basically consists of three turbinates protruding into the nasal cavity.^2^

The nasal valve is comprised of four structures. Two components are anatomical: the angle formed between the upper lateral cartilage and the septum, and the lateral diameter of the pyriform aperture. Two components are mucovascular: the head of the inferior turbinate, which is an erectile tissue, as well as the mucous tissue of the caudal septum, located dorsally to the inferior turbinate. Narrowing of the pyriform aperture and congestion of the erectile tissue of the lateral wall, especially of the inferior turbinate, associated with septal deviations, determine resistance to nasal airflow.^3^

**Figure 1:**
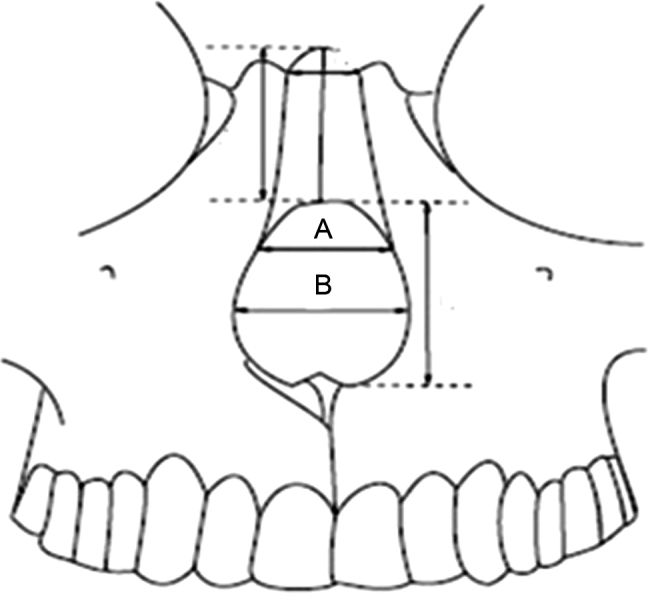
(A) Measurement of the pyriform aperture at the junction of the nasal bones with the frontal maxillary process. (B) Greatest transverse diameter of the pyriform aperture.

In a study that analyzed 88 noses of Korean individuals, mean values of 30.1 mm were found for men and 28 mm for women, transversally at the level of the pyriform aperture. The shape and size of the pyriform aperture exert a significant impact on the nasal breathing effectiveness. The size and shape of the nasal bones and the pyriform aperture can be used to clarify the anthropological characteristics of each race. The pyriform aperture of the Korean race is larger than that of the white race (Fig. 1).^4^

The treatment of nasal obstruction attributed to nasal valve dysfunction is typically aimed at interventions addressed to the internal or external nasal valve component. A 2015 study indicates that these patients can attain respiratory function improvement through surgical correction at the level of the pyriform aperture. Of twenty-six patients undergoing pyriform plasty surgery, 23 (88%) reported a significant improvement in their nasal obstruction bilaterally. The other 3 (11.5%) had a less significant improvement. The result of this study was obtained subjectively through a self-administered questionnaire. In this study, measurements using CT scan showed values of 23.6–25.32 mm in men and 22.6–23.7 mm in women.^5^

Relief of nasal obstruction through partial removal of the maxillary nasal process may improve respiratory symptoms, dry mouth and throat, exclusive mouth breathing, posterior rhinorrhea, cough and irritation of the pharyngeal mucous membrane, voice alterations due to absence of nasal resonance, headache, pressure sensation in the eyes, and loss of taste.^6^

Because the severity of nasal obstruction symptoms is not well correlated with nasal obstruction measurements, it is important to record accurate measurements of the physiological nasal obstruction. Objective methods to obtain measures of nasal resistance and patency include rhinomanometry (RM) and acoustic rhinometry (AR). These two diagnostic methods provide important information of the nasal airway. In general, RM provides information on nasal airway flow and resistance, while AR shows anatomical section areas of the nasal cavity that may be decreased.^7^, ^8^

The internal nasal valve (INV) is defined as the caudal portion of the upper lateral cartilage and also by the angle formed between the latter and the quadrangular cartilage. Laterally it consists of fibro-adipose tissue that joins the pyriform aperture, where accessory cartilage can be found. Medially, the INV is delimited by the nasal septum. Inferiorly, it is delimited by the premaxilla and posteriorly, by the head of inferior turbinate. The external nasal valve (ENV) is described as caudal structures to the INV, such as the nasal wing and ligaments juxtaposed to the lateral crus of the lower lateral cartilage (LLC), medially delimited by the columella, and inferiorly by the nostril floor.^9^

The surgical technique used to treat bone stenosis of the pyriform aperture was first described by Douglas^6^ in 1952. This pyriform aperture bone resection technique can be used in combination with rhinomanometry, which can be used to differentiate whether the nasal obstruction is essentially mucous by performing the test before and after the use of a nasal topical decongestant. The objective test is used in the quantitative evaluation of the benefit of drug and surgical therapies. The test can be used to evaluate the effectiveness of septoplasty and/or turbinoplasty in the treatment of nasal obstruction. In nasal physiology studies, rhinomanometry provides quantitative information on nasal mucosa response and changes of this mucosa in response to allergens and other types of chemical and physical stimuli.^10^

Nasal pressure is usually measured in Pascal (Pa). Pascal is the international standard unit and it is a very small unit. A pressure of 100 Pa is equal to 1 cm in height in the water column. Nasal airflow is usually measured in units of cubic centimeters per second (cm^3^/s).

Rhinomanometry is potentially the best method for objective measurement of nasal airflow obstruction, being very useful for the selection of patients who are candidates for septoplasty or nasal valve reconstruction.^11^

## Methods

From April 2015 to April 2016, eight patients screened at the Otorhinolaryngology Outpatient Clinic were selected for the study. The study was submitted and approved by an Ethics Committee under Opinion number 796.464.

Patients of both genders, older than 16 years, presenting with nasal obstruction that improved with Cottle maneuver, were included in the study. All these patients were submitted to subjective evaluation by the NOSE questionnaire. Then, they were submitted to an objective evaluation of nasal airflow through rhinomanometry, using an Atmos Rhinomanometer 300^®^ equipment, initially without the use of nasal vasoconstrictor and then using the vasoconstrictor Oxymetazoline 0.5 mg/mL at a dose of 100 μg, or two applications, followed by another application after 5 min, totaling 150 μg, according to the Committee report on standardization of rhinomanometry resolution.^12^

Patients with comorbidities that formally contraindicated any surgical procedure, patients with ulcero-granulomatous diseases and sinonasal tumors, those previously submitted to nasal surgery, and/or those with a caudal nasal septal deviation were excluded.

### Surgical technique

1. Patient placed under local anesthesia with 2% Lidocaine with Adrenaline 1: 200,000 and sedation with Propofol 200 mg/20 mL (2,6-diisopropylphenol) at a dose of 1.5–2.5 mg/kg/dose.
2. Antisepsis with chlorohexidine digluconate 2%.
3. Marking of the incision in the upper gingival-labial groove bottom (Fig. 2).

**Figure 2:**
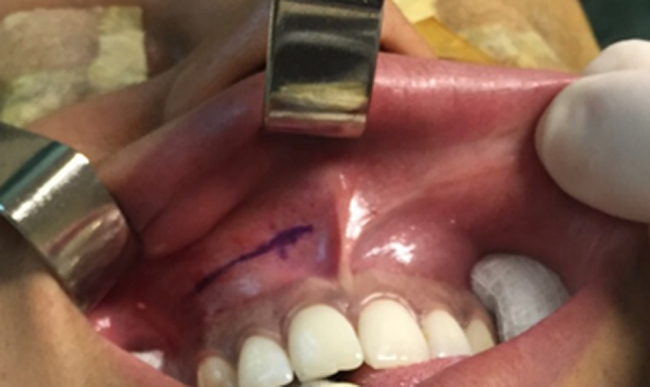
Marking of the incision in the upper gingival-labial groove bottom.
4. A 1.5 cm incision at the bottom of the R and L upper gingival-labial groove in the nasal pyriform aperture, from the central incisor to the canine on each side, preserving the lip frenulum, using a surgical blade number 15.
5. Divulsion by nasal planes and hemostasis using an electrocautery.
6. Subperiosteal detachment at the level of the pyriform aperture, including the nasal floor (Fig. 3).

**Figure 3:**
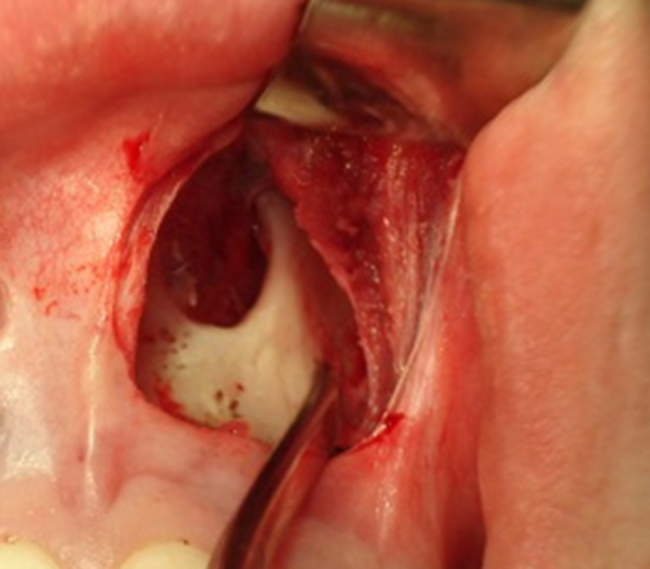
Subperiosteal detachment of the left pyriform aperture.
7. Measurement and marking of the bone portion to be removed (Fig. 4).

**Figure 4:**
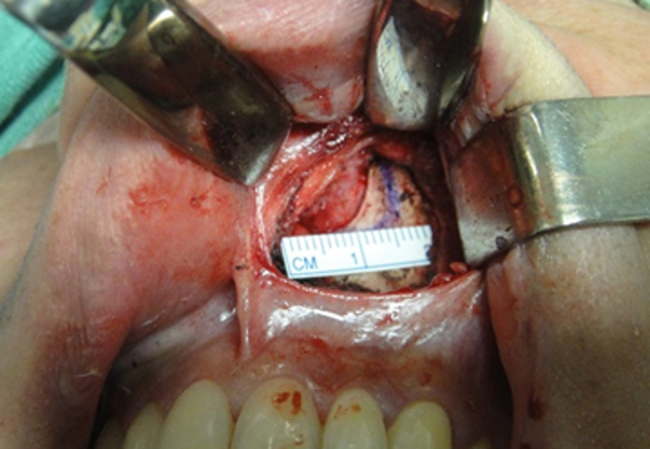
Marking the bone portion to be removed.
8. Bone removal using a 4-mm diameter steel drill, on the lateral wall of the pyriform aperture (4-mm erosion), under irrigation with 0.9% saline solution. The final result is exemplified in Fig. 5.

**Figure 5:**
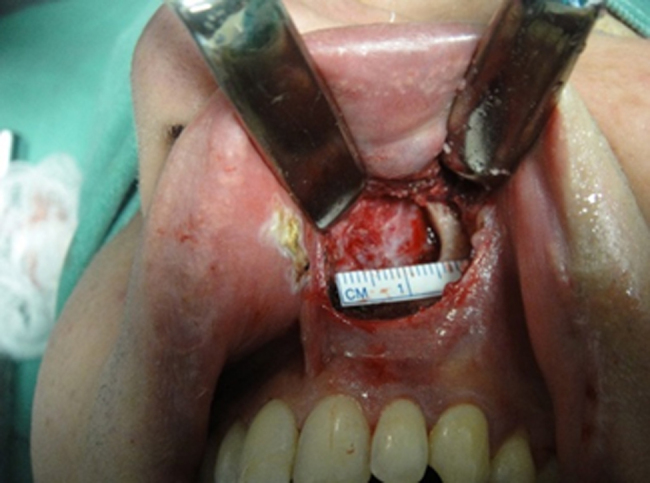
Left inferior-lateral portion of the pyriform aperture after removal with drill.
9. Suture by nasal planes with Catgut 3.0.
10. No nasal dressing or packing was used.
11. The patients were re-evaluated 7 days after the surgery.

Three months after being submitted to surgery, the patients returned and answered the NOSE questionnaire, to the same examiner, and were submitted to postoperative rhinomanometry with the same equipment, with and without vasoconstrictor agent.

## Results

The data collected through the NOSE questionnaire were analyzed quantitatively, and each patient score varied from 0 to 100, with zero score being attributed to the patient with a completely free nasal flow, without any obstruction, whereas the score 100 was attributed to the patient who had a completely obstructed nose. The results are shown in Table 1.

**Table 1:** Results of the NOSE Questionnaire in the pre- and postoperative of pyriform plasty.

	Preoperative	Postoperative
Case 1	85	70
Case 2	70	55
Case 3	55	55
Case 4	80	15
Case 5	90	25
Case 6	85	60
Case 7	75	35
Case 8	75	45

Result in number of points, ranging from zero to 100 points.

The Atmos Rhinomanometer 300^®^ device provides nasal airflow results at the pressure levels of 75, 150 and 300 Pascal (Pa) using the device's own software. These values obtained through the measures of nasal flow and airflow resistance comprise a graph showing the curves for each case, obtained in the right and left nostrils.

The rhinomanometry results in pressures of 75 Pa, 150 Pa and 300 Pa in each nostril before and after the use of nasal vasoconstrictor before and after the pyriform surgery are shown in Table 2, Table 3, Table 4.

**Table 2:** Pre- and postoperative results of nasal airflow measurements by rhinomanometry, with and without nasal vasoconstrictor, in the R and L nostrils, under 75 Pa pressure.

		Without vasoconstrictor		With vasoconstrictor	
		Pre-op flow (cm^3^/s)	Post-op flow (cm^3^/s)	Pre-op flow (cm^3^/s)	Post-op flow (cm^3^/s)
Case 1	R Nostril	324	372	384	512
	L Nostril	180	304	204	340

Case 2	R Nostril	212	280	332	380
	L Nostril	180	140	372	252

Case 3	R Nostril	184	252	276	236
	L Nostril	264	268	380	344

Case 4	R Nostril	228	128	280	248
	L Nostril	224	380	352	416

Case 5	R Nostril	204	336	292	336
	L Nostril	16	128	96	152

Case 6	R Nostril	212	220	396	456
	L Nostril	160	160	376	364

Case 7	R Nostril	148	184	424	524
	L Nostril	200	196	252	276

Case 8	R Nostril	196	124	180	324
	L Nostril	260	452	288	326

**Table 3:** Pre- and postoperative results of nasal airflow measurements by rhinomanometry, with and without nasal vasoconstrictor, in the R and L nostrils, under 150 Pa pressure.

		Without vasoconstrictor		With vasoconstrictor	
		Pre-op flow (cm^3^/s)	Post-op flow (cm^3^/s)	Pre-op flow (cm^3^/s)	Post-op flow (cm^3^/s)
Case 1	R Nostril	528	536	540	724
	L Nostril	244	428	416	480

Case 2	R Nostril	456	388	472	584
	L Nostril	240	208	556	420

Case 3	R Nostril	252	348	408	516
	L Nostril	408	388	556	1000

Case 4	R Nostril	316	280	416	376
	L Nostril	316	560	536	588

Case 5	R Nostril	224	424	440	484
	L Nostril	12	196	172	292

Case 6	R Nostril	312	324	556	720
	L Nostril	248	248	564	512

Case 7	R Nostril	220	264	620	1000
	L Nostril	280	256	364	556

Case 8	R Nostril	252	276	288	468
	L Nostril	380	348	384	460

**Table 4:** Pre- and postoperative results of nasal airflow measurements by rhinomanometry, with and without nasal vasoconstrictor, in the R and L nostrils, under 300 Pa pressure.

		Without vasoconstrictor		With vasoconstrictor	
		Pre-op flow (cm^3^/s)	Post-op flow (cm^3^/s)	Pre-op flow (cm^3^/s)	Post-op flow (cm^3^/s)
Case 1	R Nostril	696	716	736	1024
	L Nostril	328	600	636	664

Case 2	R Nostril	612	560	640	908
	L Nostril	308	264	704	624

Case 3	R Nostril	340	516	532	1000
	L Nostril	568	1000	768	1000

Case 4	R Nostril	488	420	556	520
	L Nostril	468	1000	1000	840

Case 5	R Nostril	372	808	600	684
	L Nostril	20	244	256	384

Case 6	R Nostril	468	488	716	1000
	L Nostril	340	384	1000	700

Case 7	R Nostril	316	464	832	1000
	L Nostril	404	388	500	556

Case 8	R Nostril	296	380	360	432
	L Nostril	508	872	560	688

Statistical analysis was performed using the Wilcoxon method for the NOSE questionnaire results before and after the pyriform plasty, as well as for the nasal airflow results evaluated by rhinomanometry at pressures of 75 Pa, 150 Pa and 300 Pa in the pre- and postoperative periods (Table 5, Table 6, Table 7, Table 8).

**Table 5:** Results of the Wilcoxon test applied to the pre- and postoperative pyriform plasty, considering the NOSE questionnaire, improvement with statistical relevance.

Pair of variables	*n*	Mean	Standard deviation	Min.	Max.	Percentile 25	Percentile 50 (Median)	Percentile 75	Sig. (*p*)
NOSE pre	8	76.88	11.00	55.00	90.00	71.25	77.50^a^	85.00	0.018^b^
NOSE post	8	45.00	18.71	15.00	70.00	27.50	50.00^a^	58.75	

*p*, level of significance; Min., minimum; Max., maximum.

a Results of the NOSE and pre- and postoperative rhinomanometry with nasal airflow increase in all cases at percentile 50 (median).

b Statistically significant results.

**Table 6:** Results of the Wilcoxon test applied to the pre- and post-operative pyriform plasty, evaluated by rhinomanometry under 75 Pa pressure.

Pair of variables	*n*	Mean	Standard deviation	Min.	Max.	Percentile 25	Percentile 50 (Median)	Percentile 75	Sig. (*p*)
[Pa75-wt/v] pre-op flow [r]	8	213.50	50.68	148.00	324.00	187.00	208.00^a^	224.00	0.483
[Pa75-wt/v] post-op flow [r]	8	237.00	90.91	124.00	372.00	142.00	236.00^a^	322.00	
[Pa75-wv] pre-op flow [r]	8	320.50	79.96	180.00	424.00	277.00	312.00^a^	393.00	0.036^b^
[Pa75-wv] post-op flow [r]	8	377.00	111.55	236.00	524.00	267.00	358.00^a^	498.00	
[Pa75-wt/v] pre-op flow [l]	8	185.50	78.24	16.00	264.00	165.00	190.00^a^	251.00	0.108
[Pa75-wt/v] post-op flow [l]	8	253.50	118.83	128.00	452.00	145.00	232.00^a^	361.00	
[Pa75-wv] pre-op flow [l]	8	290.00	101.80	96.00	380.00	216.00	320.00^a^	375.00	0.327
[Pa75-wv] post-op flow [l]	8	308.75	81.01	152.00	416.00	258.00	333.00^a^	359.00	

*p*, level of significance; wt/v, without vasoconstrictor; wv, with vasoconstrictor; r, right nostril; l, left nostril; P, Pascal; Min., minimum; Max., maximum.

a Results of NOSE and pre- and postoperative rhinomanometry with nasal airflow increase in all cases at Percentile 50 (median).

b Statistically significant results.

**Table 7:** Results of the Wilcoxon test applied to the pre- and post-operative pyriform plasty, evaluated by rhinomanometry under 150 Pa pressure.

Pair of variables	*n*	Mean	Standard deviation	Min.	Max.	Percentile 25	Percentile 50 (Median)	Percentile 75	Sig. (*p*)
[Pa150-wt/v] pre-op flow [r]	8	320.00	113.58	220.00	528.00	231.00	282.00^a^	421.00	0.263
[Pa150-wt/v] post-op flow [r]	8	355.00	92.40	264.00	536.00	277.00	336.00^a^	415.00	
[Pa150-wv] pre-op flow [r]	8	467.50	103.88	288.00	620.00	410.00	456.00^a^	552.00	0.017^b^
[Pa150-wv] post-op flow [r]	8	609.00	199.10	376.00	1000.00	472.00	550.00^a^	723.00	
[Pa150-wt/v] pre-op flow [l]	8	266.00	120.63	12.00	408.00	241.00	264.00^a^	364.00	0.497
[Pa150-wt/v] post-op flow [l]	8	329.00	126.05	196.00	560.00	218.00	302.00^a^	418.00	
[Pa150-wv] pre-op flow [l]	8	443.50	137.69	172.00	564.00	369.00	476.00^a^	556.00	0.141
[Pa150-wv] post-op flow [l]	8	538.50	207.31	292.00	1000.00	430.00	496.00^a^	580.00	

*p*, level of significance; wt/v, without vasoconstrictor; wv, with vasoconstrictor; r, right nostril; l, left nostril; P, Pascal; Min., minimum; Max., maximum.

a Results of NOSE and pre- and postoperative rhinomanometry with nasal airflow increase in all cases at Percentile 50 (median).

b Statistically significant results.

**Table 8:** Results of the Wilcoxon test applied to the pre- and post-operative pyriform plasty, evaluated by rhinomanometry under 300 Pa pressure.

Pair of variables	*n*	Mean	Standard deviation	Min.	Max.	Percentile 25	Percentile 50 (Median)	Percentile 75	Sig. (*p*)
[Pa300-wt/v] pre-op flow [r]	8	448.50	145.59	296.00	696.00	322.00	420.00^a^	581.00	0.123
[Pa300-wv] post-op flow [r]	8	544.00	147.42	380.00	808.00	431.00	502.00^a^	677.00	
[Pa300-wv] pre-op flow [r]	8	621.50	145.33	360.00	832.00	538.00	620.00^a^	731.00	0.017^b^
[Pa300-wv] post-op flow [r]	8	821.00	240.71	432.00	1024.00	561.00	954.00^a^	1000.00	
[Pa300-wt/v] pre-op flow [l]	8	368.00	168.18	20.00	568.00	313.00	372.00^a^	498.00	0.042^b^
[Pa300-wt/v] post-op flow [l]	8	594.00	321.81	244.00	1000.00	294.00	494.00^a^	968.00	
[Pa300-wv] pre-op flow [l]	8	678.00	251.17	256.00	1000.00	515.00	670.00^a^	942.00	0.889
[Pa300-wv] post-op flow [l]	8	682.00	183.15	384.00	1000.00	573.00	676.00^a^	805.00	

*p*, level of significance; wt/v, without vasoconstrictor; wv, with vasoconstrictor; r, right nostril; l, left nostril; P, Pascal; Min., minimum; Max., maximum.

a Results of the NOSE and pre- and postoperative rhinomanometry with nasal airflow increase in all cases at percentile 50 (median).

b Statistically significant results.

## Discussion

The nose is physiologically very dynamic; volume alterations of its structures occur at all times and, therefore, we prefer to perform several tests on the same patient, so that we can reach a conclusion and a more accurate functional diagnosis.

Regarding the subjective analysis, performed through the questionnaire answered by our patients regarding quality of life (NOSE), in cases 1 and 2 the patients showed a slight improvement in their responses, and case 3 did not mention changes, maintaining the same index in the pre- and postoperative periods. As for cases 4, 5, 6, 7 and 8, the patients reported a marked improvement in nasal obstruction. The excellent results, with a marked improvement in nasal airflow, may be justified because these patients had nasal airflow obstruction that was more dependent on the nasal valve, mainly of the bone portion, that is, narrowing of the pyriform aperture.

The rhinomanometry is currently the most objective test for evaluation of nasal respiratory function. It should be used in accordance with the ISCR (International Standardization Committee on Nasal Airway Evaluation-1984).^12^ For this reason, we chose rhinomanometry as an objective method for the evaluation of our patients.

Using the rhinomanometry assessment, four comparisons were made at each pressure level, 75 Pa, 150 Pa and 300 Pa, considering the right and left nostrils, before and after vasoconstrictor use, and before and after undergoing pyriform plasty. Therefore, we obtained a total of 12 measurement comparisons for each patient.

According to Cole^3^ in 2003, both the narrowing of the pyriform aperture and the congestion of the lateral wall erectile tissue, especially of the inferior turbinate, associated with septal deviations, determine nasal airflow resistance. This fact was observed in our results, where all patients showed nasal airflow improvement after the use of vasoconstrictors, before and after undergoing pyriform plasty.

According to Battacharyya and Deschler,^2^ the inferior turbinate is at a few millimeters from the pyriform aperture and, therefore, we believe that the increase in the diameter of the pyriform aperture promotes an area increase in this region of the inferior turbinate head, as well as in the region of the nasal wing, represented by the lower lateral cartilage.

**Figure 6:**
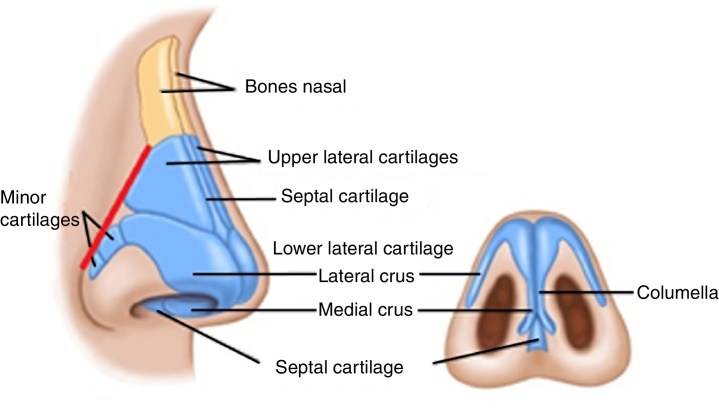
In red, the intersection of ULC and LLC with the pyriform aperture.

Based on the reports of Bhattacharyya and Deschler,^2^ and Rohrich et al.,^13^ we can postulate that, due to the connection between the upper lateral cartilage and the pyriform aperture, after the pyriform plasty the ULC and the LLC should be positioned more laterally and increase the angle formed between the ULC and the nasal septum, that is, promote nasal airflow increase through the INV. As the ligament of Rohrich et al.^13^ is a fibrous connective structure that joins the lateral crus of the LLC to the pyriform aperture, it is expected that after the pyriform plasty, Rohrich's ligament be joined more laterally to the enlarged pyriform aperture, also promoting nasal airflow increase at the level of the ENV (Fig. 6).

Spielmann et al.^14^ stated that each patient will have an indication of a specific technique, more appropriate for each case. We agree with this assertion and we believe that each case of nasal obstruction will require one or more associated surgical techniques to attain a good result, with pyriform plasty being one of them.

The Cottle maneuver can specify whether the nasal obstruction occurs at the level of the nasal valve area. This same maneuver was used in this study as one of the inclusion factors for the selection of patients who would be candidates for pyriform plasty (nasal obstruction with positive Cottle maneuver). Eventually, the best results of nasal airflow after pyriform plasty might be more pronounced if associated with other nasal surgical procedures, septoplasty and turbinectomy, for instance.^14^, ^15^ We agree with these authors.

Associated surgical procedures and clinical treatments should be considered, as there was improvement in almost all cases after nasal vasoconstrictor use, demonstrating the presence of nasal mucosa edema.

Patients who have pyriform aperture atresia associated with ogival palate with bilateral crossbite, will benefit from surgically-assisted palatine disjunction. Those with maxillary atresia, without crossbite, have an indication for pyriform plasty. Both techniques promote the enlargement of the piriform aperture.^16^

If we analyze all rhinomanometry measures, both positive and negative, at an inspiratory pressure level of 75 Pa in all patients, in both nostrils, with and without vasoconstrictor, we observe a positive value of 1434 cm^3^/s, which after being divided by 32 analyses, four in each patient, results in a measure of 44.81 cm^3^/s of increase on average. Under the pressure of 150 Pa, we obtained an increase of 2676 cm^3^/s, which, after being divided by 32 analyses, shows an average of 83.62 cm^3^/s of increase. Under the pressure of 300 Pa, we obtained a total increase of 4200 cm^3^/s, which divided by 32 measures in the 8 patients, results in an average increase of 131.25 cm^3^/s in each measure. It was concluded that nasal airflow improves more after pyriform plasty as the inspiratory pressure increases, for instance, in physical exercise.

Statistical analysis using the box plot charts showed that the median (50th percentile) was always increased postoperatively in relation to the preoperative period. With 75 Pa of resistance in the right nostril, with vasoconstrictor, nasal flow increase was observed, with a statistically significant difference. Statistically significant differences also occurred with 150 Pa of resistance in the right nostril with vasoconstrictor, and with a 300 Pa of resistance, statistically significant differences were observed in the right nostril, with vasoconstrictor, and in the left nostril without vasoconstrictor.

The NOSE questionnaire results also showed a decrease in values with a statistically significant difference.

This study should be followed by further research, aiming to support this thesis, including other pre-and postoperative evaluation techniques, which may justify other investigations, such as evaluations with CT and volumetric measurement of the nasal cavity through an appropriate software.

## Conclusion

There was nasal airflow improvement after the pyriform plasty, when compared to the preoperative nasal airflow.

## Conflicts of interest

The authors declare no conflicts of interest.
